# The Impact of Social Support on Health Outcomes of Diabetic Patients: A Systematic Review

**DOI:** 10.7759/cureus.67842

**Published:** 2024-08-26

**Authors:** Zaid Almubaid, Zachrieh Alhaj, Omar Almosa, Morad Marikh, Waliyah Khan

**Affiliations:** 1 John Sealy School of Medicine, University of Texas Medical Branch, Galveston, USA; 2 Endocrinology, Edward Via College of Osteopathic Medicine, Monroe, USA; 3 Medical School, University of Texas at Arlington, Arlington, USA

**Keywords:** patient adherence, diabetes self-management, health outcomes, social support, diabetes

## Abstract

Type 2 diabetes mellitus (T2DM) is a common chronic disease worldwide. The prevalence of T2DM has consistently increased over the past few decades. Factors affecting patient management of diabetes are important in preventing diabetic complications. Social support has been cited as one of the most important aspects of managing chronic conditions. This systematic review aims to consolidate the existing literature discussing the impact of social support on managing type 1 diabetes mellitus (T1DM) and T2DM. To begin our review, the Ovid MEDLINE (Medical Literature Analysis and Retrieval System Online) database was searched for all current literature on social support and diabetes health outcomes. Articles were then included and removed according to specific inclusion and exclusion criteria, and a systematic review was performed on the remaining articles. Twenty-two papers that met the inclusion and exclusion criteria were selected, and after data collection, a significant correlation was found between social support and health outcomes of diabetic patients and most articles reported that social support improves the health outcomes of diabetic patients. Studies show that there is some correlation between social support and improved health outcomes for diabetic patients. Further studies should be done to determine the exact correlations between social support and T2DM management and to explore the long-term impacts of social support on health outcomes for diabetic patients.

## Introduction and background

Type 2 diabetes mellitus (T2DM) is a prevalent chronic condition, with an estimation by the International Diabetes Federation (IDF) of 537 million cases worldwide [1]. The number of people with T2DM has consistently increased over the past few decades and is projected to reach 643 million by 2030 and 783 million by 2045 [2]. While genetics, obesity, and a sedentary lifestyle have been identified as risk factors for acquiring T2DM [3], factors affecting management are also important in preventing complications from the condition such as microvascular complications ranging from diabetic nephropathy, retinopathy, neuropathy, and macrovascular complications including cardiovascular disease, stroke, or peripheral vascular disease [4].

Social support has been cited as one of the most important aspects of managing chronic conditions and may play a crucial role in managing type 1 diabetes mellitus (T1DM) and T2DM. This can be defined by patient-provider relationships, familial assistance, and access to community resources [5]. These factors can influence adherence to lifestyle changes and medications, and can ultimately prevent diabetic complications, leading to psychological well-being and an improved quality of life. Numerous studies have investigated the relationship between social support and diabetes health outcomes, suggesting that social support can improve outcomes through effective management [6]. However, the extent and nature of this association remain unclear.

This systematic review aims to consolidate the existing literature discussing the impact of social support on managing T1DM and T2DM through lifestyle modifications, medication adherence, and ultimately, the impact on quality of life. This review provides a critical analysis of the evidence of this association to focus on the effective utilization of social support, identify gaps in the literature, and offer further research to provide patients and physicians with tools to successfully manage the increase in the prevalence of diabetes.

## Review

Methods

The search words used to search the Ovid MEDLINE (Medical Literature Analysis and Retrieval System Online) database were: delivery of health care, diabetes mellitus complications, diabetic outcomes, and social support. Search terms were created using boolean operators. The search resulted in 1301 papers. There was one duplicate, which was removed, resulting in 1300 articles eligible to be screened. The initial screening was based on the papers’ relevance to the topic. Papers were removed if they were not on social support and its impact on diabetic health outcomes.

**Figure 1:**
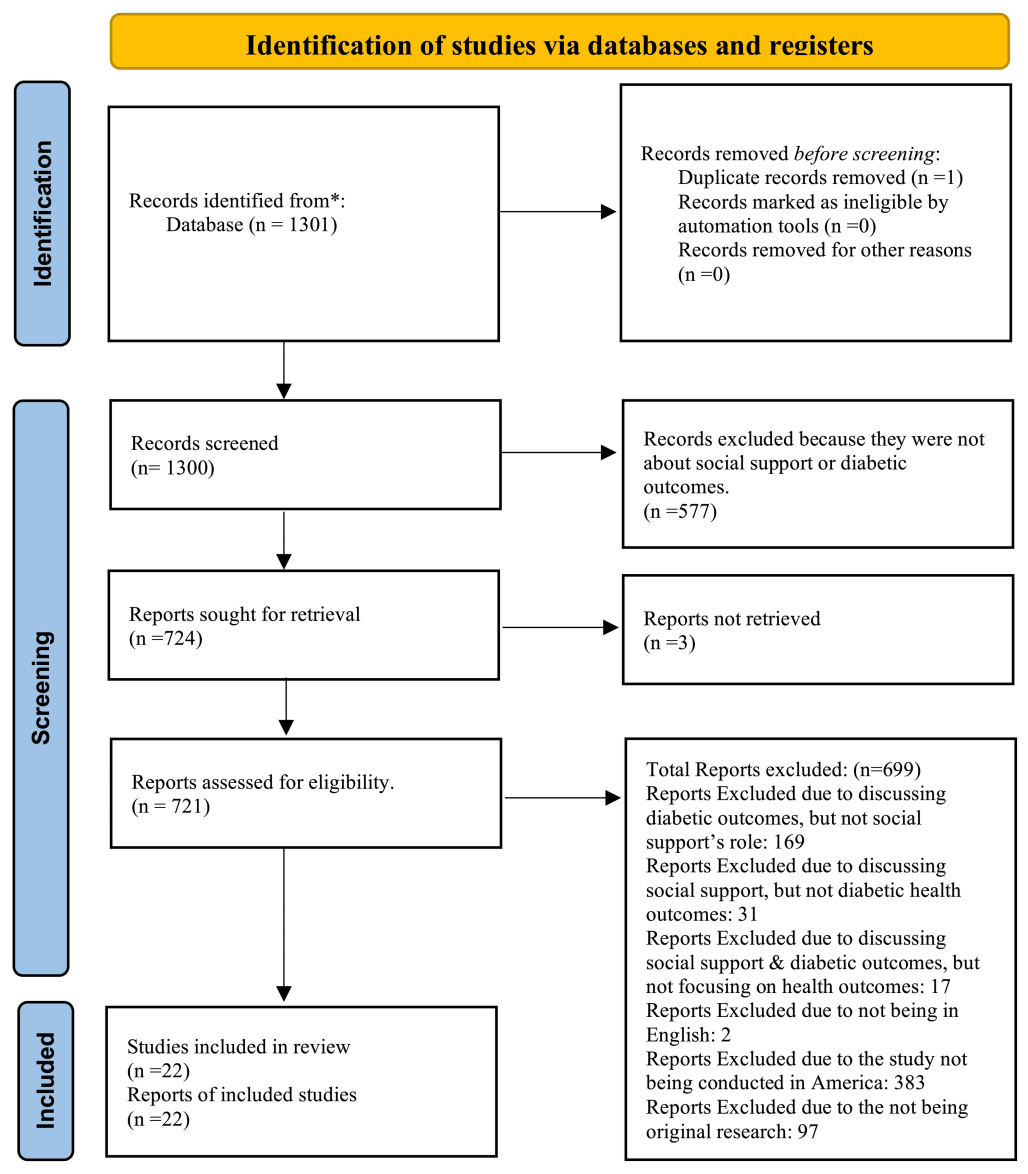
PRISMA flowchart showing the selection process of articles PRISMA: Preferred Reporting Items for Systematic Reviews and Meta-Analyses

After the initial review, 724 papers were left for review, three of which were not retrievable. The remaining 721 articles were reviewed and assessed based on our inclusion and exclusion criteria. Our inclusion criteria included any studies exploring the association between social support and diabetes health outcomes, written in English, and conducted within the United States. Exclusion criteria included papers that did not focus on social support's impact on health outcomes (169 articles excluded), did not focus on diabetes outcomes (31 articles excluded), talked about social support and diabetic outcomes but not specific health outcomes (17 articles excluded), were not written in English (two articles excluded), were not produced in the United States (383 articles excluded), and that did not contain original data (97 articles excluded). This led to a final number of 22papers that fit both the inclusion and exclusion criteria. The articles were consolidated, and limitations and potential future research were assessed, discussed, and recorded. Figure 1 is a visual representation of the selection process using the Preferred Reporting Items for Systematic Reviews and Meta-Analyses (PRISMA) model. Table 1 summarizes the findings of each study included in this review. 

**Table 1:** Summary of each study included in this review T1DM: type 1 diabetes mellitus; T2DM: type 2 diabetes mellitus

Article Title	Authors	Year Published	Study Summary
A qualitative evaluation of racial disparities in glucose control. [7]	Hannah E Shacter, Judy A Shea, Ehimare Akhabue, Naveen Sablani, Judith A Long	2009	This study evaluates the effectiveness of racially tailored interventions in reducing barriers to care and improving glucose control for African American veterans with T2DM.
A Qualitative Study of Perceived Barriers to Management of Diabetes Among Women with a History of Diabetes During Pregnancy [8]	Sarah A Collier, Celene Mulholland, Jennifer Williams, Patricia Mersereau, Khadija Turay, Christine Prue	2011	This study identifies and addresses the barriers to diabetes management in pregnant patients with pregestational or gestational diabetes to improve health outcomes for both the mother and infant.
A rural diabetes support group [9]	DB Morris	1998	This study evaluates the impact of diabetes support groups led by advanced practice nurses on improving glycemic control and self-management behaviors among diabetic patients in rural areas by addressing gaps in patient knowledge and enhancing social support systems.
An ecological perspective on diabetes self-care support, self-management behaviors, and hemoglobin A1C among Latinos [10]	Sandra C Soto, Sabrina Y Louie, Andrea L Cherrington, Humberto Parada, Lucy A Horton, Guadalupe X Ayala	2015	This study examines the influence of healthcare and family support on the self-management behaviors and hemoglobin A1C levels of Hispanic patients with T2DM living in rural areas.
An empirical study of self-efficacy and social support in diabetes self-management: implications for home healthcare nurses [11]	Caralise W Hunt, Joan S Grant, David A Pritchard	2012	This study investigates the impact of social support and education level on self-efficacy and self-management in patients with T2DM.
Associations between having an informal caregiver, social support, and self-care among low-income adults with poorly controlled diabetes. [12]	Erin D Bouldin, Ranak B Trivedi, Gayle E Reiber, Ann-Marie Rosland, Julie B Silverman, James Krieger, Karin M Nelson	2017	This cross-sectional study assesses the impact of social support and the presence of an informal caregiver on health outcomes and adherence to self-care activities in patients with poorly managed diabetes.
Associations of structural and functional social support with diabetes prevalence in U.S. Hispanics/Latinos: results from the HCHS/SOL Sociocultural Ancillary Study [13]	Linda C Gallo, Addie L Fortmann, Jessica L McCurley, Carmen R Isasi, Frank J Penedo, Martha L Daviglus, Scott C Roesch, Gregory A Talavera, Natalia Gouskova, Franklyn Gonzalez, Neil Schneiderman, Mercedes Carnethon	2015	This study examines the link between social support and health outcomes in Hispanic patients with diabetes, focusing on the correlation between low social support and premature mortality.
Contribution of family social support to the metabolic control of people with diabetes mellitus: A randomized controlled clinical trial [14]	Lilian Cristiane Gomes, Anna Claudia Martins Coelho, Danielle Dos Santos Gomides, Maria Cristina Foss-Freitas, Milton César Foss, Ana Emilia Pace	2017	This study evaluates the effects of familial social support and patient education on health outcomes for diabetes mellitus patients, utilizing Diabetes Conversation Maps to facilitate ongoing and interactive family discussions about the condition.
Evaluation of an online education and support intervention for adolescents with diabetes [15]	David B Nicholas, Karlee D Fellner, Marcia Frank, Margo Small, Ross Hetherington, Ruth Slater, Denis Daneman	2012	This study assesses the impact of online education and social support on the management and day-to-day life of adolescents with T1DM.
Factors that influence diabetes self-management in Hispanics living in low socioeconomic neighborhoods in San Bernardino, California [16]	Edelweiss Ramal, Anne Berit Petersen, Kathie Marlene Ingram, Andrea Marie Champlin	2012	This study examines the factors influencing self-management of diabetes in Hispanic patients, focusing on access to resources, dietary challenges, self-efficacy, and social support.
Family and Couple Variables Regarding Adherence in Type 2 Diabetes Patients in the Initial Stages of the Disease [17]	M Graça Pereira, Susana Pedras, Gabriela Ferreira, José C Machado	2019	This study examines how family and couples dynamics influence treatment adherence in patients with T2DM, emphasizing the crucial role of familial support in promoting behaviors like blood sugar monitoring and maintaining a healthy diet.
Family and friend participation in primary care visits of patients with diabetes or heart failure: patient and physician determinants and experiences [18]	Ann-Marie Rosland, John D Piette, HwaJung Choi, Michele Heisler	2011	This study investigates the influence of social support on healthcare outcomes for patients with chronic conditions by surveying both patients and providers, highlighting the positive effects of increased companion visits on patient satisfaction and provider-patient interactions.
Family and youth factors associated with health beliefs and health outcomes in youth with type 1 diabetes [19]	Whitney M Herge, Randi Streisand, Rusan Chen, Clarissa Holmes, Anil Kumar, Eleanor Race Mackey	2012	This study examines how family and youth factors impact health outcomes in adolescents with T1DM, emphasizing the critical role of parental involvement and family support in enhancing adherence to care plans and improving self-efficacy, thereby promoting better long-term disease management and glycemic control in youth with T1DM.
Family inclusion in diabetes education: a nationwide survey of diabetes educators [20]	Sharon A Denham , Lezlee J Ware, Holly Raffle, Kimberly Leach	2011	This study examines the influence of family inclusion in diabetes management, finding that educators' perceptions of family values significantly affect the extent of family involvement in diabetes education sessions, with potential benefits for enhancing patient self-management behaviors.
Family stress and resources: potential areas of intervention in children recently diagnosed with diabetes [21]	W F Auslander, J Bubb, M Rogge, J V Santiago	1993	This study examines the relationship between family stress, coping resources, and disease-related knowledge on glycemic control in children with T1DM, aiming to inform social work interventions that support families and improve healthcare outcomes.
I get by with a little help from my family and friends: adolescents' support for diabetes care [22]	A M La Greca, W F Auslander, P Greco, D Spetter, E B Fisher Jr, J V Santiago	1995	This study investigates the contributions of family and friends in managing diabetes in adolescent patients, emphasizing how families support medical tasks while friends provide emotional normalization.
Life stress and social support in diabetes: association with glycemic control [23]	L S Griffith, B J Field, P J Lustman	1990	This study analyzes the relationship between glycemic control, social support, and life stress in adult patients with T2DM, aiming to understand their combined impact on health outcomes.
Peer and family support in children and adolescents with type 1 diabetes [24]	Jennifer Shroff Pendley, Lori J Kasmen, Deborah L Miller, Jessica Donze, Connie Swenson, Grafton Reeves	2002	This study examines the differential effects of peer and family support on children and adolescents with T1DM, assessing hemoglobin A1C levels, perceptions of social support.
Relationship between health locus of control, health value, and social support and compliance of persons with diabetes mellitus [25]	E A Schlenk, L K Hart	1984	This study aims to explore how social support and healthcare locus of control affect self-management behaviors in Type 2 Diabetes (T2D) patients, emphasizing their compliance with treatment protocols based on health beliefs.
Supportive and nonsupportive family behaviors: relationships to adherence and metabolic control in persons with type I diabetes [26]	L C Schafer, K D McCaul, R E Glasgow	1986	The study is investigates how family support, measured by the Diabetes Family Behavior Checklist, impacts glycemic control, treatment adherence (including glucose testing, diets, and insulin injections), and dietary compliance in adolescent and adult patients with Type 1 Diabetes (T1D), examining differences between age groups and the effects of positive versus negative family behaviors.
The social context of managing diabetes across the life span [27]	Deborah J Wiebe, Vicki Helgeson, Cynthia A Berg	2016	This review explores how social support, including family, peers, significant others, and healthcare providers, influences the self-management of diabetes, particularly in children with T1DM.
Understanding the social factors that contribute to diabetes: a means to informing health care and social policies for the chronically ill [28]	Jacqueline Hill, Marcia Nielsen, Michael H Fox	2013	This paper explores how physical and social environments, such as low income, employment status, and education level, significantly influence diabetes management, emphasizing the need for innovative care delivery models to mitigate health disparities among socioeconomically disadvantaged individuals.

Results

A total of 22 papers were included in the systematic review that fit the inclusion/exclusion criteria. The studies all discussed the different ways social support can aid in improving the health outcomes of diabetic patients. The studies focused on varying forms of social support and how social support can impact the management of diabetes among patients. A summary of each study is given below.

The study by Shacter et al. discusses the use of racially tailored interventions to minimize the barriers to access to care for veterans with T2DM [7]. According to them, African Americans with T2DM tend to have lower glucose control compared to White Americans. Across all participants, African American veterans reported a higher difficulty with self-care relating to their diabetes. For both White and African American veterans, positive psychosocial factors were found to increase their glucose control. African American veterans reported a higher rate of dissatisfaction with their healthcare experience.

Uncontrolled diabetes in pregnant patients can lead to a variety of negative healthcare outcomes. Collier et al., in their study, focused on the behaviors and attitudes of pregnant patients with either pregestational diabetes or gestational diabetes and found that five common barriers made diabetes management more difficult [8]. These include financial barriers, communication barriers, lack of social support, inability to maintain a healthy diet, and barriers impeding diabetes treatment. They stated that addressing these barriers to care is essential, as this can improve health outcomes for both the mother and infant.

According to Morris [9], support groups can help bridge the gap between providers and patients living in rural areas who are physically distant from their healthcare providers. Diabetes support groups allow participants to share their experiences with the chronic condition, gain knowledge about management, and improve their level of glycemic control. The community health nursing project implemented by Morris et al. aimed to educate diabetic patients living in rural areas. Advanced practice nurses identified gaps in patient knowledge regarding diabetes, and then led support groups to address the gaps that they identified. The overall effectiveness of support groups, according to Morris, can be attributed to the relationship between social support and healthcare outcomes. It was found that patients with adequate support systems participated in more self-management behaviors to control and treat their diabetes.

The study by Soto et al. focused on Hispanic patients with T2DM living in rural areas [10]. Hemoglobin A1C levels were measured in patients while analyzing their self-management, social, and organizational practices. Both healthcare and family support positively affected the patients' A1C levels. Other self-supporting behaviors were seen at a higher rate when social and healthcare support was positive and present. These behaviors included eating more fruits and vegetables, exercising more, and consuming less high-fat foods. This finding highlights the importance of support from both healthcare providers and the patient's family. Thus, the more support a T2DM patient receives, the more likely they are to adhere to treatment and management protocols.

Management of T2DM can be supplemented by external forms of social support. According to Hunt et al., determining how these social support factors affect the self-management of this chronic condition is crucial [11]. In general, they found that those with high self-efficacy were more capable of self-managing their T2DM. External support can promote one’s self-efficacy, which in turn will increase the ability to manage T2DM. Hunt et al.'s study concluded that higher education level was associated with increased self-efficacy in T2DM patients and those with sufficient social support could manage their diabetes more effectively.

Bouldin et al.'s cross-sectional study aimed to determine whether the presence of social support and an informal caregiver affects health outcomes in patients with poorly managed diabetes [12]. They monitored three self-care activities in patients with and without informal caregivers: eating a healthy diet, performing foot checks, and continuous blood glucose monitoring. They found that those with an informal caregiver were more likely to complete these tasks than those without an informal caregiver. Their study defined an informal caregiver as a family member or friend who assists with the treatment or management of diabetes. In addition, patients with an informal caregiver were seen to be more likely to adhere to medication schedules than those without.

According to the study by Gallo et al., the Hispanic population is at an elevated risk of developing diabetes, and they found that low social support is associated with premature mortality in Hispanic patients with diabetes [13]. Their study discussed the “Hispanic Paradox”, where Hispanic patients appear to have a lower risk of CVD morbidity and increased lifespan relative to non-Hispanic White patients. Possible explanations for this paradox were discussed, but there was no definite evidence to support this finding.

Gomes et al., in their study, aimed to determine the effects of familial social support on health outcomes for patients with diabetes mellitus [14]. Educating the patient and their families on topics relating to diabetes was done to initiate conversations surrounding this chronic condition. Diabetes Conversation Maps were used to direct the conversation among the patient and their families. Their study found that patient education should be implemented in a gradual, continuous, and interactive manner.

The study by Nicholas et al. tried to determine the importance of education and support for adolescents with T1DM [15]. Education was provided online, where participants were able to learn about others' experiences with T1DM as well as gain knowledge regarding the management of this chronic condition. Social support was found to affect how well adolescents with T1DM managed their chronic condition. Patients noted that many aspects of their day-to-day lives had been altered due to having T1DM. According to the results of the study, having social support and effective interventions can help adolescents gain a sense of normalcy when managing their diabetes.

Ramal et al. discussed the factors that influence the self-management of diabetes in Hispanic patients in their study [16]. The four main factors were: access to resources, struggling with diet, self-efficacy, and social support. For social support to effectively increase health outcomes in Hispanic diabetic patients, they found that family and friends should be educated on the treatment and management of diabetes. Their study discussed the importance of educating the patient and their families, and how this can lower the risk of diabetes for future generations.

Pereira et al.'s study addressed the role of relationship dynamics in the adherence to treatment in patients with T2DM [17]. The effects of family and couple dynamics were assessed one year after diagnosis with T2DM, and then again four months later. It was found that patients were more likely to monitor their blood sugar and maintain a healthy diet if they were supported by their family or significant other. The factors that were essential for the effective management of T2DM were family coping and marital adjustments. Their study highlighted the importance of including the patient's family in the treatment and management of T2DM.

According to Rosland et al., the degree of social support that a patient receives can impact how they manage their chronic condition [18]. They surveyed both patients and providers to determine the effects of social support on healthcare outcomes in patients with chronic conditions. It was found that increased visits from companions led to a more positive experience for both the patients and the providers. Companions were observed to provide emotional support and assistance with day-to-day tasks. It was also found that patients were more satisfied with their experience with their primary care physician when they were accompanied by a family member or friend.

Herge et al. found that the management of T1DM in adolescents becomes easier when the patient is surrounded by a sufficient social support system [19]. Their study focused on the relationship between family/youth factors and the subsequent effects on health outcomes in youth with T1DM. It was found that parental involvement played a very crucial role in the management of the child’s T1DM. Patients were more likely to adhere to care plans if support from their family was evident. It was also found that sufficient family support increases self-efficacy. This in turn leads to better disease management in the long run, ultimately leading to better glycemic control in youth with T1DM.

The role of family inclusion in the management of diabetes was analyzed in the study by Denham et al. [20]. Certified diabetes educators completed surveys to determine the importance of family inclusion in the management of diabetes. It was found that the educators’ understanding of family values influenced how much they included the patient's family in diabetes education sessions. By educating patients and their families with family theory in mind, there could be a significant increase in self-management behaviors in diabetic patients.

The health status of a child with T1DM can directly impact the way that their family deals with stress according to the study by Auslander et al. [21]. Their study aimed to examine the level of family stress, coping, and resources that were available. It was found that higher levels of family stress and less access to resources were directly correlated to poor glycemic control. By determining this connection between familial support and healthcare outcomes, they suggest that social work interventions could be implemented to better support families and patients attempting to manage T1DM. Disease-related knowledge was also measured in both the child with T1DM and their parents.

The role of family and friends in the management of an adolescent patient with diabetes was analyzed by La Greca et al. [22]. Patients’ families tended to provide more support in the management of the chronic condition, which included giving insulin shots, monitoring the patient’s blood glucose, and providing healthy meals. Friends were more likely to provide emotional support, allowing the patient to “normalize” their experience with diabetes. It was found that the younger the patient was, the more familial support they received. In addition, treatment adherence was increased with more familial support. The findings of this study highlight the importance of social support in the management and treatment of diabetes in adolescent patients.

The relationship between glycemic control and social support for patients with T2DM was analyzed in the study by Griffith et al. [23]. A third factor of life stress was also analyzed in adult T2DM patients. Although previous research found a significant correlation between social support, stress, and glycemic control, this study was not able to find a significant correlation between these factors. Instead, the researchers found that patients with high life stress and low social support were more likely to experience health complications.

According to the study by Pendley et al., peer and family support have differing effects on children and adolescents with T1DM [24]. Children between the ages of eight and 17 years were surveyed to determine hemoglobin A1C levels, perceptions of social support, and self-reported management measures. Adolescents (aged 12-17 years) with T1DM reported more peer support related to their disease management compared to younger children aged 7-11 years. There was no significant correlation found between family support and hemoglobin A1C levels, which represented the degree of metabolic control.

According to the study by Schlenk and Hart, social support and the healthcare locus of control directly impacted self-management behaviors in patients with T2DM [25]. Compliance with diabetes treatment and management protocols was found directly related to patients' health beliefs. This study found that patients with low health motivation will poorly manage their diabetes. In addition, it was found that those with adequate social support, and an internal health locus of control were more compliant regarding continuing diabetes management.

Family support can have different impacts on adolescent and adult patients with T1DM as per the study of Schafer et al. [26]. Family support behaviors were measured by the Diabetes Family Behavior Checklist. Adolescents showed a lower level of glycemic control compared to adult patients. However, family support was more evident in adult patients, who were more likely to adhere to glucose testing, diets, and insulin injections due to familial support. Both positive and negative family behaviors led to higher dietary compliance. In adolescents, there was no significant correlation to predict treatment adherence.

Self-management of diabetes is one of the most important factors in managing the disease. However, social support is another factor that can enhance a patient’s motivation to independently manage their diabetes. The social resources such as the patient's family, peers, significant others, and healthcare providers were analyzed in the study by Wiebe et al. to determine their impact on the patient's management of diabetes [27]. It was found that behaviors such as acceptance, collaboration, and warmth were associated with better management. T1DM is a condition that is commonly diagnosed in children who are not fully capable of self-managing their diabetes. In this study, it was found to be especially important for families to play a role in management and treatment to encourage positive health outcomes.

The influence of a patient’s physical and social environments on their management of diabetes was discussed by Hill et al. [28]. Factors of the physical and social environment include low income, employment status, and low education level. These factors can negatively impact health outcomes in patients with diabetes. Hill et al. addressed diabetes as a public health issue and the role of social determinants in T2DM maintenance. It was found that the prevalence of T2DM was “socially graded”, meaning that those with lower income and education levels experienced poor health outcomes. To address the socially graded nature of diabetes, according to the authors, innovative models of care delivery are needed to better educate and treat those of a lower socioeconomic class. 

Discussion

For adequate treatment and management of diabetes, patients are encouraged to adhere to their medications and make lifestyle changes to improve their health outcomes. Our review found that increased social support does aid in the improvement of health outcomes of diabetic patients. Whether the social support is familial, via education, or improved access to resources, our review found that diabetic patients will have better outcomes. In addition, our review has found that a decrease in social support, or lack thereof, has led to worse health outcomes among diabetic patients [29]. 

The literature shows that when the Affordable Care Act was enacted, patients more readily had access to treatment and had also improved access to valuable resources for patients with diabetes in the Hispanic community which led to improved health outcomes in these patients [30]. When family members or a patient's social support becomes more involved, these family members play an important role in increasing said patients' medication adherence. Lifestyle changes such as an improved diet for these patients were also found upon family member involvement in the patient’s treatment [12,17,26]. Improved diabetes health outcomes were also observed when the patient felt accepted in their family or their social support systems, allowing the patient to feel less alienated because of their diabetes [27]. This acceptance came through diabetes education to the patient’s social support, which was repeatedly shown in the literature to help in the treatment of diabetes [15,20].

To improve treatment in diabetes patients, it is important to delve into areas of research that showcase the most effective ways to increase social support [21-26]. Most studies in this review highlighted the importance of education for both the patient and the members of the social support system [9-28]. From this, we know that improving health outcomes is reliant on better education about diabetes for the patient and the families, and methods that effectively deliver education to patients as well as their support system should be further researched to identify if they lead to long-term improved health outcomes in patients with diabetes.

## Conclusions

In the current review, improved social support was seen to be associated with improved health outcomes in diabetic patients. When the patient had improved access to resources, familial support, and acceptance in the community, their diabetes was proven to be more controlled. With patient and familial education about diabetes being one of the forefront factors that studies have highlighted to improve social support, more research is required on effective ways to deliver education to patients and its long-term impact on the health of diabetic patients.
